# Tissue-resident lymphocytes: sentinel of the transformed tissue

**DOI:** 10.1186/s40425-017-0244-3

**Published:** 2017-05-16

**Authors:** Saïda Dadi, Ming O. Li

**Affiliations:** Immunology Program, Memorial Sloan Kettering Cancer Center, 1275 York Avenue, New York, NY 10065 USA

**Keywords:** Cancer immunosurveillance, Innate lymphoid cells, Innate-like T cells

## Abstract

Tumor cells can be detected and cleared by lymphocytes in a process termed cancer immunosurveillance. However, the contributing cell types had not been fully characterized. Using oncogene-induced murine models of epithelial cancer, a recent study showed that cell transformation triggers expansion of tissue-resident lymphocytes derived from innate, T cell receptor (TCR) αβ and TCRγδ lineages. These type-1-like innate lymphoid cells (ILC1ls) and type 1 innate-like T cells (ILTC1s) share a gene expression program distinct from those of conventional lymphocytes, and exhibit cytolytic activities against tumor cells. Further deciphering such a tumor-elicited immunosurveillance mechanism may 1 day be harnessed for novel cancer immunotherapy.

## Text

Innate lymphocytes function as sentinels of infection and tissue distress by sensing changes in the environment. They respond by expressing an array of inflammatory mediators that shape subsequent immune responses, and/or induce target cell cytotoxicity. For example, conventional natural killer (cNK) cells, the prototypical member of the innate lymphoid cell (ILC) family, sense cellular stress signals as well as the missing self, and participate in host defense responses against viral infection and some types of cancer [1]. Our recent study has revealed that solid tumors of epithelial origin activate and expand a group of type 1 lymphocytes derived from innate, TCRαβ and TCRγδ lineages [2]. In spite of differential expression of the T cell receptor, these lymphocytes share the characteristics of being tissue-resident, exhibiting cytotoxic activities against tumor cells, and displaying a common gene expression program distinct from those of cNK cells and conventional T cells. The tumor-associated type 1 innate lymphocytes were named type 1-like innate lymphoid cells (ILC1ls) since they are transcriptionally related but functionally distinct from type 1 innate lymphoid cells (ILC1s), and are transcriptionally distinct but functionally related to cNK cells. The tumor-associated type 1 T cells are distinct from conventional CD8 T cells or invariant NKT cells, but are highly similar to ILC1ls, and were named type 1 innate-like T cells (ILTC1s). The ILC1l and ILTC1 response is sustained through the stage of tumor development, and unlike conventional CD8 T cells, ILC1ls and ILTC1s do not express the T cell exhaustion marker PD1.

Unlike conventional lymphocytes that are recruited from the blood to the site of insult, many innate lymphocyte populations possess a distinct feature of being tissue-resident [3]. Group 1 ILCs have been shown to play a critical role in maintaining local tissue homeostasis and their dysregulation can lead to immunopathology. For example, a recent study demonstrated that ILC1s, but not cNK cells, induces organ dysfunction in a mouse model of ischemic kidney injury [4]. Another study showed that adipose tissue-resident ILC1s contribute to obesity-related immunopathology [5]. Our study revealed that innate lymphocytes expand early in the transformed tissue in response to yet-to-be defined signals. The tissue residency property of ILC1ls and ILTC1s likely makes them the first-line responders of cell transformation. To this end, it is interesting to note that similar to tissue-resident memory CD8 T cells, NKT cells and liver-resident ILC1s, ILC1ls and ILTC1s express high levels of the transcription factor Gm13060 (also known as Hobit), which has recently been shown to direct a transcriptional program of tissue residency [6]. Future studies will determine whether Hobit plays an essential role in driving the tissue resident program of ILC1ls and ILTC1s.

Group 1 ILCs, including cNK cells and ILC1s, are characterized by their high expression of the transcription factor T-bet and the activating receptors NKp46 and NK1.1 in mice [7]. cNK cells and ILC1s are different lineages of innate lymphocytes, but are both dependent on Nfil3 for differentiation [8–10]. In addition, *Nfil3* ^−/−^ mice have reduced numbers of the common helper-like innate lymphoid progenitor (CHILP) indicating that Nfil3 promotes the generation of all lineages of ILCs [11]. CHILP-derived ILCs are plastic and adapt to the cytokine milieu [12], suggesting that the tumor-associated ILC1ls could differentiate from cNK cells or CHILP-derived ILCs. Nevertheless, despite high expression of T-bet, NKp46 and NK1.1, the tumor-elicited ILC1l response is largely intact in the absence of Nfil3. Notably, the salivary gland (SG) ILC1s are independent of Nfil3 either [13]. Both cell types are found in glandular tissues and express CD103, raising the possibility that such tissue microenvironment may overcome Nfil3 deficiency to promote the generation and/or maintenance of these cells. Alternatively, ILC1ls may differentiate following a developmental pathway distinct from those of cNK cells and ILCs, and they are selectively seeded in glandular tissues.

Based on the expression of CD127 and CD103, human ILC1s have recently been divided into two populations [14]. Unlike CD127^+^ ILC1s that can be converted to ILC3s by polarization cytokines, CD103^+^ ILC1s are recalcitrant to such conversion, implying that CD103^+^ ILC1s and CD127^+^ ILC1s are distinct subsets [14]. A recent report has identified a circulating precursor population with the potential of generating ILCs and NK cells in tissues suggesting a non-medullary mechanism of human innate lymphocyte differentiation [15]. In addition, another recent report has revealed a subset of CD3^−^CD56^+^ innate lymphoid cells in human ovarian cancer [16]. How mouse ILC1ls are related to the diverse populations of human type 1 innate lymphocytes, and their exact cell differentiation pathways await future characterization.

ILTC1s are tissue-resident T lymphocytes expressing a diverse repertoire of T cell receptors; yet, these cells possess innate cytolytic activities against tumor cells. High expression of CD103 and several NK cell receptors on ILTC1s are reminiscent of those on intestinal epithelial lymphocytes (IELs) and skin-resident TCRγδ T cells. Recent studies have revealed that innate-like T cells might be selected by agonist antigens in the thymus, and their TCR signaling is suppressed upon maturation concomitant with acquisition of innate lymphocyte characteristics [17]. These findings raise the intriguing possibility that ILTC1s may deviate from the conventional lineage of T cells following their agonistic selection in the thymus and attenuation of TCR signaling in the periphery. Defining the selection antigens for ILTC1s and the mechanisms of signal rewiring will clarify the ontogeny of ILTC1s and how they are related to other innate-like T cell subsets. Furthermore, it remains to be determined whether TCR expression is only functional during ILTC1 thymic selection or it has an additional role in the periphery. Whether ILC1ls or ILTC1s have complementary or redundant activities need be clarified as well. Recent studies have suggested that ILCs and T cells are part of a multilayered immune organization in which they perform overlapping functions, providing de facto robustness to the immune system [18]. It is therefore possible that the presence of both ILC1ls and ILTC1s in pre-cancerous lesions could strengthen the cancer immunosurveillance response.

The common gamma chain cytokine IL-15 is essential for the generation of ILC1ls and ILTC1s, and IL-15 deficiency results in accelerated tumor growth. However, it remains to be determined whether IL-15 promotes the differentiation, homeostasis, and/or activation of these cells in the transformed tissue. IL-15 is expressed in diverse lineages of cell types including macrophages, dendritic cells as well as adipocytes, fibroblasts and epithelial cells, and requires trans-presentation to function, raising the possibility that it may act as an alarmin cytokine to support immune responses against infection, cell stress or tumor formation [19]. In coeliac disease, IL-15 and the cNK cell-activating ligands are expressed on epithelial cells, which may activate IELs to induce immunopathology. To this end, it is interesting to note that tumors with high levels of IL-15 have high density of intratumoral cytotoxic T cells, and the loss of the *IL15* gene in human colon cancer is associated with poor patient prognosis [20]. These observations suggest that the transformed epithelium may provide a critical source of IL-15 to activate tissue-resident lymphocytes. Nonetheless, to delineate the exact IL-15-mediated cellular mechanisms of cancer immunosurveillance awaits the generation of cell type-specific IL-15-deficient mouse models.

Immunotherapy targeting inhibitory receptors PD1 and CTLA4 expressed on conventional lineages of T lymphocytes have demonstrated remarkable success in cancer patients [21]. However, not all patients treated with these checkpoint blockade antibodies experience tumor shrinkage, durable responses, or prolonged survival. The elucidation of tumor-induced ILC1l and ILTC1 response raises the intriguing possibility of targeting the unconventional tissue-resident lymphocytes for cancer therapy through alternative mechanisms distinct from checkpoint blockade. It is conceivable that the maximal potential of cancer immunotherapy will not be achieved until multiple facets of the host defense responses are unleashed.

## Abbreviations

CHILP: Common helper-like innate lymphoid progenitor
cNK: Conventional natural killer cells
IELs: Intestinal epithelial lymphocytes
ILC: Innate lymphoid cell
ILC1ls: Type-1-like innate lymphoid cells
ILTC1s: Type 1 innate-like T cells
SG: Salivary gland
TCR: T cell receptor
